# Growth factor-enriched autologous plasma improves wound healing after surgical debridement in odontogenic necrotizing fasciitis: a case report

**DOI:** 10.1186/1752-1947-5-98

**Published:** 2011-03-11

**Authors:** Rubi Lopez-Fernandez, Jorge Ramirez-Melgoza, Nora Ernestina Martinez-Aguilar, Alicia Leon-Chavez, Daniel Martinez-Fong, Juan Antonio Gonzalez-Barrios

**Affiliations:** 1 Departamento de Cirugía Maxilofacial, Hospital Regional "1o de Octubre", Av. IPN No. 1669, México D. F., C.P. 07760, México; 2 Laboratorio de Medicina Genómica, Hospital Regional "1o. de Octubre", Av. IPN No. 1669, México D. F., C.P. 07760, México; 3 Departamento de Fisiología, Biofísica y Neurociencias, Centro de Investigación y Estudios Avanzados, Av. IPN No. 2508, México D. F., C.P. 06760, México

## Abstract

**Background:**

Odontogenic necrotizing fasciitis of the neck is a fulminant infection of odontogenic origin that quickly spreads along the fascial planes and results in necrosis of the affected tissues. It is usually polymicrobial, occurs frequently in immunocompromised patients, and has a high mortality rate.

**Case presentation:**

A 69-year old Mexican male had a pain in the maxillar right-canine region and a swelling of the submental and submandibular regions. Our examination revealed local pain, tachycardia, hyperthermia (39°C), and the swelling of bilateral submental and submandibular regions, which also were erythematous, hyperthermic, crepitant, and with a positive Godet sign. Mobility and third-degree caries were seen in the right mandibular canine. Bacteriological cultures isolated *streptococcus pyogenes *and *staphylococcus aureus*. The histopathological diagnosis was odontogenic necrotizing fasciitis of the submental and submandibular regions. The initial treatment was surgical debridement and the administration of antibiotics. After cultures were negative, the surgical wound was treated with a growth factor-enriched autologous plasma eight times every third day until complete healing occurred.

**Conclusions:**

The treatment with a growth factor-enriched autologous plasma caused a rapid healing of an extensive surgical wound in a patient with odontogenic necrotizing fasciitis. The benefits were rapid tissue regeneration, an aesthetic and a functional scar, and the avoidance of further surgery and possible complications.

## Introduction

Cervical necrotizing fasciitis (CNF) is an uncommon, rapidly progressive, and potentially lethal infection comprising skin, subcutaneous tissue, superficial fascia, and occasionally the deep fascia. Its rapid progression results in necrosis and severe systemic toxicity. The incidence of CNF is 2.6% out of the infections of head and neck [1]. Clinical manifestations include pain and local erythema. The skin turns dark with purple dots. The pressure on the zone reveals gas accumulated by the excessive metabolism of bacteria. In advanced stages, thrombosis of local blood vessels of the skin and subcutaneous tissue leads to necrosis and later to gangrene. This infectious pathology is common in people who use drugs and/or alcohol, people with diabetes, immunocompromised individuals, and patients with pressure ulcers. The conventional management consists of a vigorous debridement of necrotized skin, subcutaneous tissue, all fascias, and muscle, along with specific antimicrobial treatment. Once the affected area is free from any infection, the surgical defect is treated using restorative plastic surgery that involves the rotation of a pedicle or a skin graft [2].

Growth factors (GFs) are biomolecules that regulate a great variety of key functions in the body, including mitosis, cell differentiation, extracellular matrix synthesis, and metabolism. During the ontogeny, some GFs also display chemotactic activity to direct cell migration. Several families of GFs are expressed in specific tissues where they play a protective role against the natural and pathologic cell death. Generally, the production and the physiological activity of GFs occur at low concentration (picomolar, pM) in a wide variety of cells. All these advantages have supported the use of GFs in different medical procedures. Several GFs can be obtained at different concentrations from peripheral blood to provide their biological actions on a particular tissue or organ with a lesion. In wound healing, GFs significantly decrease the time of tissue regeneration [3] and scarring by improving cell metabolism, causing protein synthesis, and promoting cell proliferation [4].

In this work, we report the effectiveness of GF-enriched autologous plasma to promote rapid and functional healing of an extensive surgical wound resulting from the aggressive surgical debridement in a patient with odontogenic necrotizing fasciitis. Because the wound comprised the submental and submandibular regions, the main goal was to provide an aesthetic and functional scar in the neck, thus avoiding secondary reconstructive plastic surgery and further complications.

## Case Report

**Figure 1:**
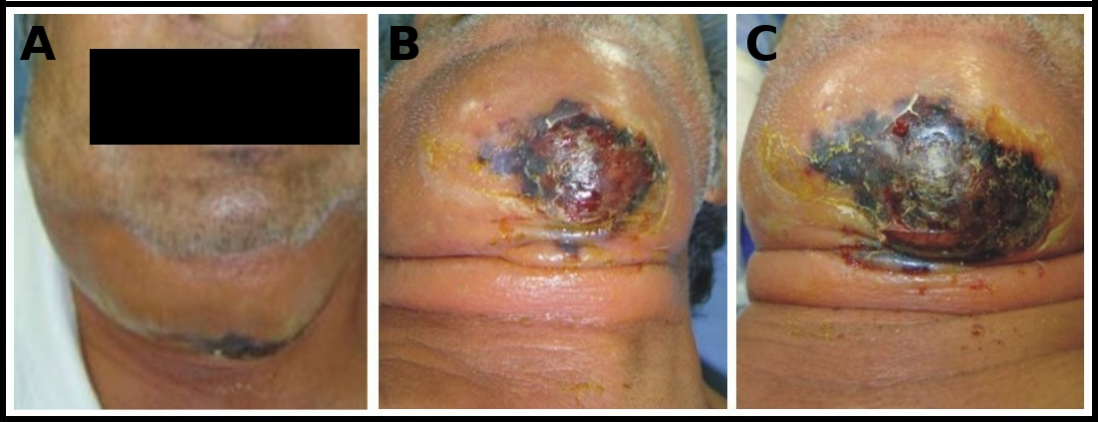
**Male patient with an odontogenic necrotizing fasciitis**. **A) **Necrotic area after emergency room admission, **B) **Necrotic area after three-hour evolution. **C) **Necrotic area after eight-hour evolution.

A 69-year-old Mexican male (Figure 1 panel A) was referred to the Maxillofacial Surgery Department of the "Hospital Regional Primero de Octubre (ISSSTE)" in Mexico City. The patient had pain in the maxillar right-canine region and a three-day swelling of the submental and submandibular regions. The clinical examination showed pain during palpation, tachycardia, and hyperthermia (39°C). The bilateral submental and submandibular regions had a 10-cm diameter swelling and well-delimitated erythematous, hyperthermia, crepitant sound, and a positive Godet sign. After a three-hour evolution, a blister surrounded by a 4-cm diameter necrotic area could be detected (Figure 1 panel B). After an eight-hour evolution, the original diameter of the necrotic area had doubled (Figure 1 panel C). An intraoral examination showed mobility and third-degree caries of the mandibular right canine. The patient had a history of diabetes mellitus type II, moderate malnutrition, chronic alcoholism, and hepatic disease. Laboratory studies provided the following relevant results: hemoglobin, 11.1 g/dL; hematocrit, 31.4%; leukocytes, 15 × 10^3^/dL of blood, whose differential count was as follows, (neutrophils, 12.72 × 10^3^; lymphocytes, 0.85 × 10^3^; monocytes, 0.83 × 10^3^; eosinophils, 0.45 × 10^3^; basophils, 0.14 × 10^3^; platelets, 78 × 10^3^); glycemia, 185 mg/dL; calcium, 6.49 mg/dL; phosphorus, 2.68 mg/dL; creatinine, 0.76 mg/dL; total protein, 5.2 mg/dL; albumin, 2.4 mg/dL; total bilirubin, 7.3 mg/dL; prothrombin time (PT), 17.2 sec. A bacteriological culture isolated *streptococcus pyogenes *and *staphylococcus aureus*. The final diagnosis was odontogenic necrotizing fasciitis of the submental and submandibular regions, which was corroborated by a trans-surgical biopsy of the affected area.

Aggressive surgical debridement was made 8 hours and 24 hours after the patient's admission. Both submandibular glandules and the suprahyoideus and infrahyoideus muscles were exposed. At this time, the dimensions of the postsurgical wound were 10.5 × 7.3 cm (area = 76.7 cm^2^). The antibiotics administered were ceftriaxone (2 g, IV, every 24 h), clindamycin (600 mg, IV, every 6 h), and amikacin (500 mg, IV, every 12 h). Consecutive bacteriological cultures were made until the results were negative. The decision for the treatment of the surgical wound with GF-enriched autologous plasma to promote tissue regeneration was made considering the systemic condition of the patient (diabetes, liver failure, normocytic anemia, hypocalcaemia, malnutrition, hyperbilirubinemia, decompensate respiratory alkalosis, and confusional syndrome).

**Figure 2:**
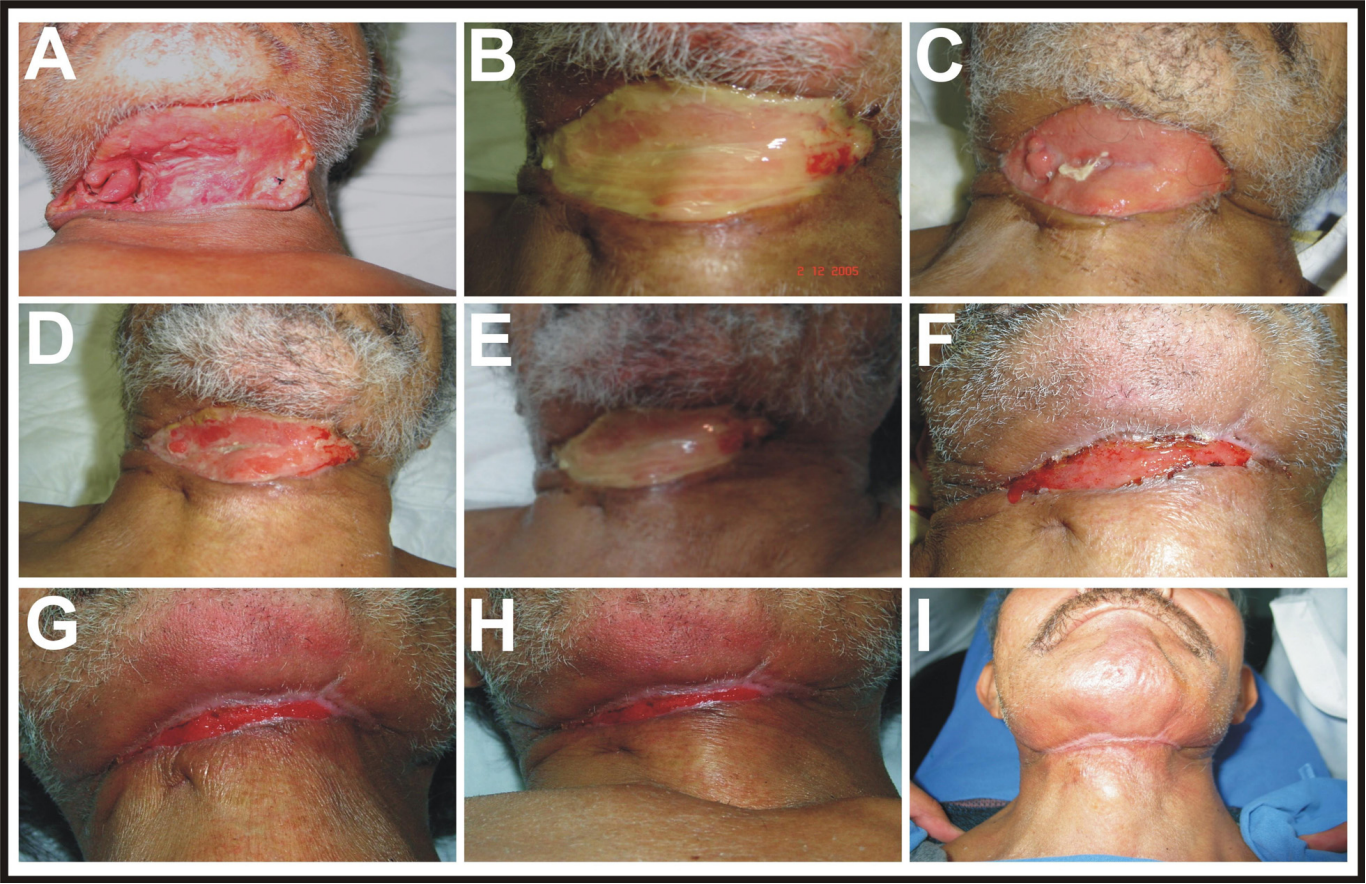
**Photographic sequence of results after GF-enriched autologous plasma application**. The photographs show the progressive decrease of the surgical defect until the entire healing of the wound. **A)** Surgical wound with negative bacteriological culture. **B)** Application of polymerized GF-enriched autologous plasma. **C-H)** Temporal course of surgical wound healing from the week 1 to the week 6. **I)** Aesthetic and functional scar at the end of the week 7.

**Figure 3:**
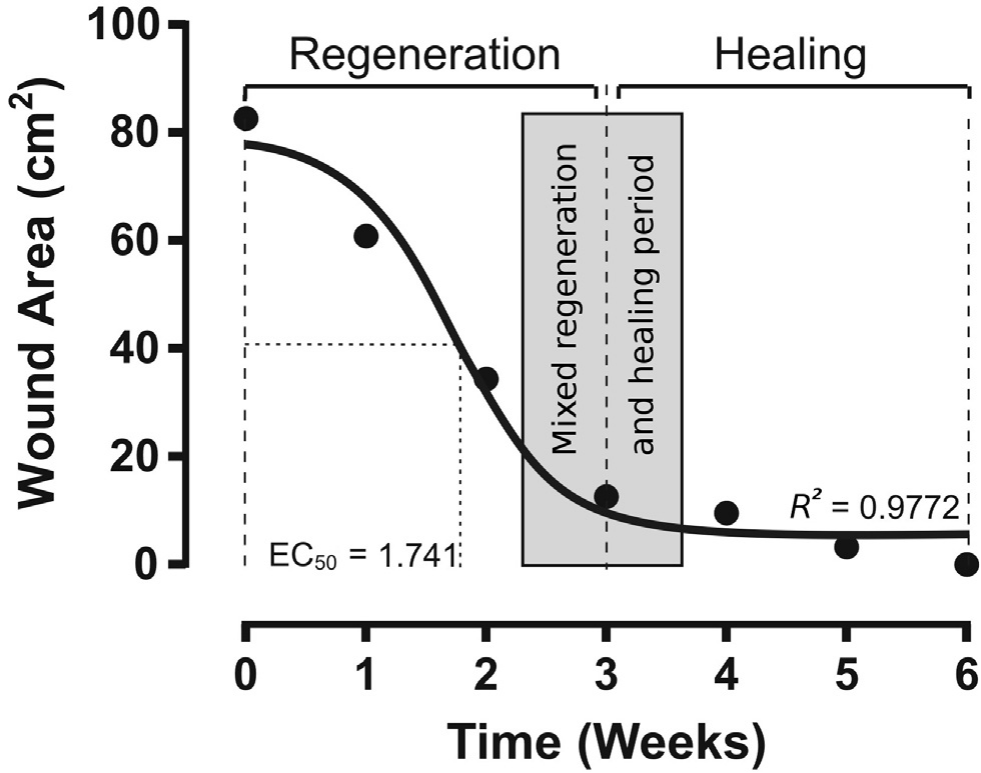
**Temporal course of surgical wound healing evaluated by the remaining debrided area**. The black circles are the measurement of the wound area. The continuous line was the curve fit using nonlinear regression analysis of the GraphPad Prism 5.0 statistical package (GraphPad Software Inc., La Jolla, CA, USA). EC_50 _is the time required to obtain 50% healing of the wound. *R^2 ^*is the coefficient of determination.

The GF-enriched plasma was obtained from 15 mL of the patient's peripheral blood as described elsewere [5,6]. This volume was divided equally into 5 sterile tubes containing 0.5 mL of 3.8% sodium citrate as an anticoagulant. After centrifugation at 1800 rpm for 8 minutes at room temperature, the plasma had divided into three fractions of about equal volume. The upper fraction is plasma poor in GFs, the middle fraction is plasma with a medium concentration of GFs, and the lower portion is plasma rich in platelets and GFs [7]. The lower portion was collected from all 5 tubes and pooled into one sterile tube. To induce platelet degranulation, fifty μL of 10% CaCl_2 _solution was added for each mL of platelets and GFs enriched plasma and the mixture was gently stirred to allow it to gel [8,9]. Finally, a total volume of 2 × mL of the gel fraction containing GF-enriched plasma, with pink-yellow color, was separated from the fraction of platelet rich plasma (translucent color) [3]. Approximately 1.5 mL of the gel fraction was directly applied in the center of the wound and then manually spread to cover the total area of the surgical wound. This procedure was repeated every third day until the completion of the wound healing (Figure 2). The local application of GF-enriched autologous plasma caused tissue repair in a considerably reduced time (Figure 3). The nonlinear analysis of the temporal course of wound healing determined the concentric regeneration period was within weeks 0 to 3 and the healing process period was within weeks 4 to 6. A mixed period of regeneration and healing extended from 15 to 25 days. The time required to achieve 50% closing of the surgical wound was 12 days and the total closure was reached at week 6 after the onset of the GF-enriched autologous plasma treatment (Figure 3).

## Discussion

This study shows for the first time the advantages of the topical use of GF-enriched autologous plasma on an extensive surgical wound in the neck of a patient with odontogenic necrotizing fasciitis caused by *streptococcus pyogenes *and *staphylococcus aureus*. Our treatment avoided the formation of a function-limiting scar, one of the most frequent complications following an aggressive surgical debridement of necrotized tissues in the neck. Remarkably, the rapid and physiological healing of the surgical wound avoided further reconstructive plastic surgery, which involves pedicled graft rotation and secondary facial deformation.

The occurrence of necrotizing fasciitis remains an extremely uncommon condition with a limited number of cases reported in the literature. The lack of medical experience makes the diagnosis difficult, which allows the infection to rapidly spread to skin, subcutaneous tissue, superficial fascia, and occasionally the deep fascia, ending up in severe necrosis and systemic toxicity [2]. The prognosis depends on an early diagnosis, nutritional support, effective wide-spectrum antibiotics, and an aggressive surgical debridement along with several surgical washes every 24 to 48 hours until bacteriological cultures are negative [10]. Our therapy with repeated topical application of GF-enriched autologous plasma on the surgical wound improved the prognosis of a patient with odontogenic necrotizing fasciitis. Our clinical experience surely will be useful in establishing a routine treatment for sterile wounds requiring rapid and physiological healing.

Other clinical reports show the efficiency of GF-enriched autologous plasma in healing wounds from different origins. We propose it as an alternative therapy to provide tissular regeneration of skin, bone, and muscle [11-13]. Although effective for tissular regeneration, the therapeutic use GF-enriched autologous plasma is still controversial because its preparation does not consistently provide the same type and concentration of GFs [7]. An initial characterization of GF-enriched autologous plasma shows the presence of platelet-derived growth factor (PDGF), transforming growth factor-ß1 (TGF-ß1), basic fibroblast growth factor (bFGF), vascular-endothelial growth factor (VEGF), epidermal growth factor (EGF), and insulin-like growth factor type I (IGF-I). All these GFs are involved in skin regeneration and scarring, because all together stimulate cell mitosis and differentiation, promote angiogenesis, granulation tissue formation, re-epithelialization, and stimulate extracellular matrix and collagen synthesis. Nevertheless, the concentration of GFs in the gel of GF-enriched autologous plasma is variable and depends on the general conditions of the patient, especially the nutritional and immunological state. This is why the therapy was the repeated topical application until the complete healing of the wound is achieved. Despite thrombocytopenia of the patient, the procedure to obtain GF enriched autologous plasma was very effective to provide the concentrations of GFs required to activate their receptors and exert their trophic effects. The ability of GF's to activate their receptors at low concentrations, in the range of picomolar (pM) to nanomolar (nM) [14] can account for the therapeutic effect. Even though we ignored the type and exact concentration of GFs released from the gel of GF-enriched autologous plasma, it is unquestionable that our therapeutic scheme provided effective concentrations of GFs. The photographic sequences (Figure 2) and the mathematic analysis (Figure 3) of the temporal course of the concentric regeneration and healing processes demonstrate the success of our therapy. The functional and neuropsychological restoration allowed the patient to promptly return to a normal family and social life.

## Conclusions

The gel of GF-enriched autologous plasma provided an aesthetic and functional scar in the neck that allowed the patient to recover his normal life in a relatively short time after treatment. The optimum neuropsychological adaptation avoided the use of further reconstructive plastic surgery and the possible development of unnecessary complications. On this basis, we propose the topical use of GF-enriched autologous plasma as a coadjuvant procedure in the management of patients with necrotizing fasciitis.

## Patient's perspective

Before the beginning of my father's treatment, photographs of other people with the same disease and treated with conventional management were shown to me. The photographed people looked deformed by the surgery. Now, when I see my father completely recovered and healthy, I think that the new treatment offered to my father was the best, and I believe in the use of this treatment for other patients with the same disease.

## Competing interests

The authors have no financial and personal relationships with other people, or organizations that could inappropriately influence their work, all within 3 years of beginning the work submitted.

## Authors' contributions

RLF and JRM treated the patient and prepared the case report. NEMA and ALCH prepared the PRGF. DMF was the scientific advisor and prepared the final version of this manuscript, and JAGB conceived the paper and prepared the figures. All authors read and approved the final draft of the manuscript.

## Consent

Written informed consent for publication of this case report and accompanying images was obtained from the patient's daughter who is his legal representative, because the patient is illiterate. A copy of the written consent is available for review by the Editor-in-Chief of this Journal.
